# Sinus Bradycardia in an Adolescent Taking Lamotrigine

**DOI:** 10.1155/2022/3353684

**Published:** 2022-05-13

**Authors:** Genevieve Davis, Kristen Ward, Sarah Mohiuddin

**Affiliations:** Department of Psychiatry, University of Michigan, USA

## Abstract

Lamotrigine is commonly prescribed for the treatment of neurological conditions and is increasingly being prescribed for psychiatric conditions as well. Although largely well tolerated, it is known to have a number of potential side effects, and in March 2021, the FDA issued its most recent warning for the medication due to its increased risk of cardiac arrhythmias. In this report, we describe a case in which an adolescent patient was found to be bradycardic after starting lamotrigine for antidepressant augmentation, with a gradual return to normal heart rate as the medication was subsequently tapered and discontinued. Further research is needed to more accurately estimate the risk of cardiac side effects and to establish appropriate monitoring guidelines for cardiac arrhythmias in those taking lamotrigine.

## 1. Introduction

Lamotrigine is a medication that is often used off-label for children and adolescents for the management of mental health concerns. It is approved by the United States Food and Drug Administration (FDA) in adult populations for the indications of epilepsy and bipolar disorder. For children and adolescents, lamotrigine has been FDA approved for the indications of epilepsy only [5]. Off-label uses of the medication include antidepressant augmentation, in the treatment of borderline personality disorder, and as a preventative agent for migraine [1, 8]. There is evidence that the rates of off-label use have been steadily increasing over time [1].

There are many known side effects from taking lamotrigine, and there are currently FDA warnings in place for the risk of life-threatening rash, suicidal thoughts, aseptic meningitis, hemophagocytic lymphohistiocytosis, hypersensitivity reactions, and blood dyscrasias, as well as the recent addition of a warning for possible cardiac arrythmias. Relatively little is known about the cardiac side effects of lamotrigine, and the FDA is now requiring that additional in vitro studies be completed to understand this risk more clearly [5]. This report details a case of sinus bradycardia in an adolescent patient who had recently been started on lamotrigine. The patient's guardians provided written informed consent for this publication.

## 2. Case Presentation

Patient E. was a 15-year-old nonbinary adolescent who had been assigned female gender at birth and now uses either she or they pronouns. She resided with her maternal grandparents, who were also her legal guardians. E. had an extensive complex trauma history which involved emotional and physical caregiver abuse, neglect, witnessed domestic violence, and sexual assault. Mood disturbance first presented in 2015, with prominent depressed mood, anhedonia, irritability, chronic wish for death, poor self-image, hypersomnia, anergia, and reduced appetite. Historically, she had also engaged in nonsuicidal self-injurious behaviors including cutting and scratching. E. reported that since the onset of depressive symptoms in 2015, she has a history of multiple suicide attempts.

Her treatment history was notable for a brief course of supportive psychotherapy to which she had minimal response, as well as previous trials of sertraline and citalopram prescribed by her primary care physician with minimal benefit. Family history was significant for polysubstance abuse in both biological parents as well as a childhood diagnosis of oppositional defiant disorder in her biological mother. She was in the 10^th^ grade and generally performed well academically, although grades had declined slightly in the previous year in the context of her worsening depressive symptoms and changes in the academic environment due to the COVID pandemic. She endorsed intermittent use of tobacco and cannabis, which she estimated occurred at an average frequency of twice weekly. She had no significant past medical history and family history was negative for cardiac concerns.

In 2020, due to worsening psychiatric symptoms and lack of response to two antidepressant trials, E. established treatment in psychotherapy, and after a few sessions, she was referred for a medication evaluation with a psychiatrist in the same clinic due to the severity of her symptoms. Diagnostically, she met criteria for major depressive disorder and posttraumatic stress disorder. During her medication evaluation in January 2021, she was switched from citalopram to fluoxetine to target both depressive and trauma-related symptoms. At her medication evaluation, it was also discovered that E. had been attempting to restrict her food intake due to body image concerns and she was requiring significant prompting from her grandparents to complete meals. She reported laxative use up to twice weekly due to constipation but denied additional purging. She was referred to the adolescent medicine clinic for further evaluation of her disordered eating. At her initial adolescent medicine visit, it was determined that she met the diagnostic criteria for anorexia nervosa given her 17-pound weight loss over a seven-month period due to restricted intake. Vitals were obtained at that visit which demonstrated a pulse of 88 beats per minute and a blood pressure of 126/63. E. and her grandmother were instructed to start having three fully supervised meals and two supervised snacks daily.

At her follow-up psychiatry visit in April, fluoxetine had been titrated to 40 mg daily with a partial positive response, although residual depressive symptoms of passive thoughts of suicide, anergia, hypersomnia, and anhedonia continued to be quite bothersome. The decision was then made to initiate adjunctive therapy. Lamotrigine was chosen as the augmenting agent due to the patient's strong preference to be on a weight neutral medication that did not require lab monitoring due to her fear of needles. Her history of disordered eating precluded the use of bupropion. Lamotrigine was started at 25 mg at bedtime, and they were instructed to increase the total daily dose by 25 mg every two weeks.

Three weeks following lamotrigine initiation, she had a follow-up appointment with adolescent medicine. Her dose of lamotrigine at that time was 25 mg in the morning and 50 mg at bedtime, and she reported a partial positive response to that dose. She and her grandmother reported significantly improved food intake over the past several weeks, and she denied any recent purging or other compensatory behaviors. Weight had remained stable from the previous visits, from 50.5 kg to 50.8 kg. Vitals obtained at that visit demonstrated a pulse of 48 beats per minute and blood pressure 113/56. EKG was obtained due to her low pulse, which showed sinus bradycardia with a ventricular rate of 50 beats per minute. E. was asymptomatic. Labs obtained at that visit including comprehensive metabolic panel, complete blood count, magnesium, phosphorus, and thyroid stimulating hormone were all within normal limits. It was not felt that E.'s bradycardia could be attributed to protein-calorie malnutrition given stable weight, normal lab work, and lack of clinical signs of malnutrition. Her adolescent medicine providers contacted her psychiatry team to notify them of the bradycardia. Due to concern that lamotrigine could be the responsible agent, the decision was made in collaboration with the clinic's pharmacist to begin tapering lamotrigine while monitoring for improvement in heart rate. Since the discovery of her bradycardia at her adolescent medicine appointment, her grandmother had been checking her vitals at home with an automated blood pressure cuff two to three times per day, and she reported that E.'s pulse was consistently in the low 50s. As lamotrigine was tapered, her heart rate gradually began increasing into the mid-50s and up to 60 beats per minute. Within three weeks of discontinuing lamotrigine, her resting heart rates had improved to the high 50s to 60s, with occasional recordings in the low 70s. E.'s food intake remained stable throughout the tapering of lamotrigine.

## 3. Discussion

This case describes an adolescent who developed bradycardia shortly after the initiation of lamotrigine as an antidepressant augmentation agent. Sinus bradycardia in a patient without a prior cardiac history appears to be a rare side effect of lamotrigine, which has never been described in the literature for either pediatric or adult populations.

Lamotrigine is approved by the FDA for the management of epilepsy in patients aged 2 years and older as an adjunctive treatment, as a monotherapy for epilepsy in patients 16 years and older, and as a maintenance treatment for bipolar disorder in adults [5]. There is some evidence to suggest that in pediatric populations off-label use of lamotrigine may provide a benefit for bipolar disorders and unipolar depressive disorders and for management of aggression [7]. Lamotrigine is considered to be a generally well-tolerated medication, with the most commonly reported side effects of nausea, headache, and mild rash being reported at rates similar to placebo [8]. However, lamotrigine does carry a low risk of more severe side effects, and the FDA has issued warnings regarding the risk of serious rash, hemophagocytic lymphohistiocytosis, hypersensitivity reactions, blood dyscrasias, suicidal behavior and ideation, and aseptic meningitis. In March 2021, the FDA issued a new warning for the risk of cardiac rhythm and conduction abnormalities, which states that lamotrigine “could cause serious arrythmias and/or death in patients with certain underlying cardiac disorders or arrhythmias” [5].

It has been hypothesized that one of lamotrigine's primary mechanisms of action of inhibiting voltage-gated sodium channels may be responsible for its cardiac effects, particularly when used at toxic levels at which it may lose specificity for neuronal sodium channels. The sodium channel SCN5a, found in cardiac myocytes, may be involved in this process [9]. Mutations in SCN5a have been associated with several conduction abnormalities including long QT syndrome, progressive cardiac conduction disease, Brugada syndrome, and sinus bradycardia [11]. Case reports have been published on Brugada syndrome and prolonged QRS complex in instances of lamotrigine toxicity [3, 9]. One report has been published on a case of refractory ventricular fibrillation in a previously healthy female taking lamotrigine [4]. A report on sudden unexpected death in epilepsy (SUDEP) in four female patients who were prescribed lamotrigine monotherapy has also been published [2]. While the question of lamotrigine contributing to a fatal cardiac arrhythmia was raised, there was also debate about whether other causes such as medication nonadherence or uncontrolled seizures may have been the more likely causes of SUDEP in these four cases [10]. Furthermore, it is possible that patients with epilepsy may have a greater baseline risk of developing arrhythmias when taking medications that act on sodium channels due to cardiocerebral channelopathy syndromes that may underlie their seizure disorder [6]. As the majority of literature reviewing the arrhythmogenic potential of lamotrigine appears to be in the context of epilepsy treatment, the risk of developing an arrhythmia while taking lamotrigine for primary psychiatric purposes is even less understood.

Possible confounding variables in the presented case include the patient's comorbid disordered eating history as well as the patient's occasional cannabis use. As was noted in the case, E.'s adolescent medicine providers had a very low suspicion that her bradycardia could be attributed to her food intake as her grandparents were providing close supervision during, and after all meals, her weight had remained stable, and her labs did not show evidence of protein-calorie malnutrition. There are case reports that cannabis can lead to slowed heart rate via parasympathetic activation at high doses [12], though the fact that E.'s cannabis use remained stable prior to, during, and following the course of her treatment with lamotrigine suggests that this was not the primary cause of her bradycardia.

The risk of cardiac effects in patients without a prior cardiac history who are taking lamotrigine at a nontoxic dose has not been established. As this case suggests, more research is needed in this area both to identify populations who are most at risk of developing cardiac side effects and to establish clear clinical guidelines on how to monitor for and manage this risk in patients taking lamotrigine for primary neurologic or psychiatric indications.

## Data Availability

Data used to support the findings of this study are included within the article.
